# The needs of the many: Exploring associations of personality with third-party judgments of public health-related utilitarian rule violations

**DOI:** 10.1371/journal.pone.0284558

**Published:** 2023-04-21

**Authors:** Alexander Behnke, Diana Armbruster, Anja Strobel

**Affiliations:** 1 Department of Psychology, Technische Universität Dresden, Dresden, Germany; 2 Institute of Psychology and Education, Ulm University, Ulm, Germany; 3 Department of Psychology, Chemnitz University of Technology, Chemnitz, Germany; Goethe University Frankfurt am Main, GERMANY

## Abstract

Safeguarding the rights of minorities is crucial for just societies. However, there are conceivable situations where minority rights might seriously impede the rights of the majority. Favoring the minority in such cases constitutes a violation of utilitarian principles. To explore the emotional, cognitive, and punitive responses of observers of such utilitarian rule transgressions, we conducted an online study with 1004 participants. Two moral scenarios (*vaccine policy* and *epidemic*) were rephrased in the third-party perspective. In both public health-related scenarios, the protagonist opted against the utilitarian option, which resulted in more fatalities in total, but avoided harm to a minority. Importantly, in *vaccine policy*, members of the minority cannot be identified beforehand and thus harm to them would have been rather *accidental*. Contrariwise, in *epidemic*, minority members are identifiable and would have needed to be *deliberately* selected. While the majority of participants chose not to punish the scenarios’ protagonists at all, 30.1% judged that protecting the minority over the interests of the majority when only accidental harm would have occurred (*vaccine policy*) was worthy of punishment. In comparison, only 11.2% opted to punish a protagonist whose decision avoided deliberately selecting (and thus harming) a minority at the cost of the majority (*epidemic*). Emotional responses and appropriateness ratings paralleled these results. Furthermore, complex personality × situation interactions revealed the influence of personality features, i.e., trait psychopathy, empathy, altruism, authoritarianism, need for cognition and faith in intuition, on participants’ responses. The results further underscore the need to consider the interaction of situational features and inter-individual differences in moral decisions and sense of justice.

## Introduction

Protecting the rights of a minority within a larger group or society is one of the cornerstones of modern democracy. However, there are situations in which upholding these rights infringes upon the rights of the majority. In moral decision making, such conditions have often been conceptualized as a conflict between *deontic* and *utilitarian* rules. While *deontic* rules are based on situation-transcending fundamental principles of right and wrong, *utilitarian* rules are outcome-based in favor of the ‘greater good’, i.e., to optimize the benefit for all [1]. Depending on the actual situation, choosing the needs of the few over the needs of the many might not result in any noticeable harm to the majority at all. However, there are situations in which the consequences might be severe and decision makers might be held accountable for wrongful harm to the majority. Research into moral judgement makes frequent use of hypothetical dilemmas [2] in which deontological and utilitarian principles tend to elicit different responses, although concerns have been raised regarding some of these dilemmas’ external validity [cf. 3, 4]. Certain dilemmas have been criticized for their lack of realism which calls their usefulness as assessment tools of moral decision-making into question [4]. Still, their simplified structure has also been argued to be useful for shedding light on the underlying structure of moral reasoning [5]. Nevertheless, these clear-cut scenarios with forced-choice answering formats do not necessarily capture ‘pure’ utilitarian resp. deontological principles. Rather, depending on the scenario, multiple ethical considerations are likely to be factored into decisions to varying degrees [e.g., 6]. Thus, a decision favoring a minority against a majority is not necessarily solely due to deontological reasoning [see also 7]. Accordingly, decisions against majority interests, which we investigated might alternatively be characterized more generally as non-utilitarian rather than deontic. Overall, despite known limitations hypothetical dilemmas remain useful for assessing various aspects of moral decision-making.

### The role of the observer

Since utilitarian rule violations potentially affect society at large, third-party responses to them are of particular interest and of theoretical as well as practical importance. Third parties or noninvolved observers are essential for successfully dealing with moral transgressions in larger societies. Increasing anonymity and loose one-time interactions make third-party punishment a necessity to ensure sufficient and consequential sanctions [8–10]. Maintaining social cooperation and interpersonal trust depend on consistent punishment of rule violations—if need be by third parties [9]. Hence, uninvolved observers have been suggested to act as everyday judges who assess observance of social norms and inflict sanctions in cases of transgressions [11]. However, to date, most research on moral decision making in dilemma situations has focused on the first-party perspective [e.g., 12–16]. Studies employing third-party perspectives often yielded similar results although differences between first- and third-party judgements have also been reported [17, 18]. In sum, despite its recognized importance there is less research on third-party decision making in moral dilemma scenarios [19, 20] particularly with regard to personality traits that might potentially influence it.

Within a broader research project, we investigated third-party judgements that aimed to explore the role of personality traits and dilemma features associated with differences in responses to (a) deontological rule violations [results published in 21] and (b) utilitarian transgressions. To examine the latter, two well-established fictional moral dilemmas [13, 14] were adapted to a third-party perspective: *vaccine policy* and *epidemic*.

### Dilemma features

In both scenarios, the respective protagonist decided against the ‘utilitarian’ option, i.e., against the needs of the many. While trying to safeguard the rights and more importantly the lives of a minority, the protagonists’ decisions ultimately resulted in more net harm. In *vaccine policy*, a fatal disease is spreading, which can be prevented by vaccinating as many people as possible. However, since a very small proportion of vaccinated people is expected to die due to being allergic to the vaccine, the person in charge decided not to impose compulsory vaccination on the population. As a result, millions died from the disease although no one died because of intolerance to the vaccine. In *epidemic*, the medicine for treating a raging deadly disease is scarce and cannot be quickly produced. Thus, not every patient can be treated on time. Due to known genetic differences, a minority (X type) requires five times more of the drug than the rest of the population (Y type). The person in charge decided against excluding members of the minority from the treatment. As a result, X-type people were also saved, however, for every X-type person saved, five other people died who could not be treated (for the exact wording of the two dilemmas see S1.1 moral scenarios in S1 File). Although one might assume that our study was done in the wake of the ongoing COVID-19 pandemic, we actually planned and conducted it before the outbreak. Investigating influence factors that shape reactions to such moral dilemmas may provide valuable insight into how the public can be expected to respond and how potential risks and benefits should be best communicated to the public.

While the two dilemmas share important features, they also differ with regard to others. In *vaccine policy*, deciding in favor of the majority (i.e., a ‘utilitarian’ decision) would have resulted in the *accidental* deaths of a minority. Prior to vaccination, it is unknown which persons are part of this risk group. Thus, they cannot be selected before vaccination. Conversely, in *epidemic*, members of the X-type minority would have had to be identified in order to be actively and *deliberately* excluded from treatment (and thus condemned to die) to save more Y-type individuals. This difference is not trivial. The dilemmas’ protagonists had to weigh saving the lives of the majority at the cost of (a) the unintentional deaths of a minority that is unidentifiable prior to the intervention (*vaccine policy*) vs. (b) the intentional and targeted exclusion of identified minority members from life-saving treatment (*epidemic*). Thus, the justifiability of opting against the utilitarian choice differs between the two scenarios.

### Moral emotions and cognitions

Aside from dilemma characteristics, additional factors are likely to modulate third-party responses to these two scenarios. Previous research confirmed that deliberate cognitions as well as intuitive emotional reactions exert complex effects on moral decisions [14, 22, 23]. Moral transgressions which result in greater harm or unfairness inflicted upon a victim elicit in third parties stronger negative emotions like contempt, disgust, anger, or disappointment towards a perpetrator [e.g., 22, 24–26]. These negative emotions are in turn associated with harsher punishment [22, 24, 27–30]. The influence of perceived unfairness or harm on punishing tendencies is thus likely to be mediated by negative moral emotions [31–33]. Although negative emotions are frequently the main focus, moral dilemmas often result in an intricate mix of partly contradictory feelings including the positive sentiments compassion, comprehension, or sympathy [25, 34, 35] that need to be taken into account. Unsurprisingly, positive emotions towards a protagonist are associated with milder punishment and weakened negative emotions [e.g., 31, 36, 37].

### Personality and moral decision-making

Previous findings indicate several personality traits like psychopathy, empathy, altruism, justice sensitivity, and authoritarianism as well as thinking styles as potential modulators of third-party judgment. Early clinical characterizations of *psychopathy* as “moral derangement” [38] and “moral insanity” [39] point to a long recognized link with deviant morality. Psychopathy is characterized by shallow affect, callousness, manipulative tendencies, lack of remorse or guilt, and antisocial behavior [40–42] and is associated with deficits in assessing the appropriateness of moral actions [42–44]. However, psychopaths’ classification of certain (mostly utilitarian) actions as ‘immoral’ has also been found to be similar to controls, but they nevertheless tended to opt for them [45–47]. This preference for utilitarian options has been suggested to be due to lack of empathic concern for potential victims [40] as well as reduced aversion towards actions, particularly towards harmful actions [42]. In a recent study of third-party responses to acts of killing, trait psychopathy was associated with reduced concern about deontic rule violations and a more marked appreciation of utilitarian motives to kill but also with harsher punishment [21]. Similarly, lower *empathy* scores have been linked to utilitarian choices in the general population [41, 48], whereas heightened empathic affect leads to the avoidance of utilitarian options [49]. In addition, psychopathy and less altruistic attitudes are associated with higher tolerance for moral transgressions [50]. Although the concepts lacks of a clear definition across disciplines [see 51 for a review], a core feature of *altruism* is the tendency to voluntarily help others while not expecting reward and putting one’s own needs second to those of others. Regarding third-party punishment, one facet of altruism is of particular interest: the costly punishment of moral violations without or with little direct benefit to those who punish [52–54]. Altruistic punishment plays a key role in maintaining cooperation [e.g., 26, 55] and in increasing equality of group members [56].

Another important trait with regard to third-party judgments is *justice sensitivity*. The trait refers to an inclination to adopt social perspectives of victims, perpetrators, observers, or beneficiaries of unjust or unfair incidents [57]. Higher justice sensitivity scores are linked to reduced trait psychopathy, more empathic concern and perspective taking, and a less lenient attitude towards transgressive behavior in moral dilemmas [41]. In third parties, adopting a victim’s perspective triggers negative empathic concern, while envisioning a perpetrator causing harm produces aversive moral emotions [49, 58]. Furthermore, increased observer justice sensitivity predicts harsher third-party punishment and stronger feelings of anger [28].

An additional trait of interest is *authoritarianism* which is characterized by compliance with and dogmatic protection of conventional (often religious) standards, an uncritical submission to authorities, and hostile sentiments as well as retaliatory actions against those who violate norms [59–61]. Regarding moral decisions, one characteristic finding is the association of authoritarianism (and the highly correlated religious fundamentalism) with a clear preference for fixed rules irrespective of situational demands [7, 62, 63]. Hence, people with an authoritarian or religiously fundamentalist mindset both tend to conform to (deontic) rules and experience enhanced negative emotions in moral dilemma situations [61, 64]. Thus, they show the opposite response pattern compared to psychopaths in terms of rule compliance and emotional reactions.

Finally, thinking styles like *need for cognition* (NFC) and *faith in intuition* [FI; 65] appear to modulate moral judgment and behavior [66]. Deliberative thinking styles like NFC have been linked to a preference for utilitarian judgments [7, 67, 68]. Furthermore, NFC was found to promote moral courage and self-reported moral behavior [66, 69, 70]. In third parties, deliberate and authority-independent thinking styles have also been linked to an increased consideration of potential utilitarian motives when judging homicides [21]. Findings on FI are less consistent, i.e., while FI was associated with a preference for deontic decisions in some studies [71], others could not confirm a link between FI and morality [7, 66].

### Study aims

As outlined above, there are several state- as well as trait-like influence factors on moral decision-making. Generally, moral choices depend on a complex interplay of cognitive and affective responses [14, 22, 23], personality aspects [cf. 21] as well as features of the situation resp. dilemma [72, 73]. Our main aims were to explore the following variables and their association with third-party judgements of utilitarian rule violations: (a) dilemma characteristics (i.e., avoidance of *accidental* vs. *deliberate/targeted* harm), (b) personality traits (i.e., psychopathy, empathy, altruism, justice sensitivity, authoritarianism, NFC, and FI), and (c) moral emotions and cognitive processes. Both investigated dilemmas describe hypothetical public health scenarios, although similar situations are nevertheless feasible in real life. Exploring how third parties respond thus also offers an insight into how populations might react to the implementation of corresponding measures in related circumstances.

## Materials and methods

We report how we determined our sample size, all data exclusions, all manipulations, and all measures in the study [cf. 74]. All materials, data, and code are openly available at https://osf.io/qhsnt/. The study was carried out as part of a larger research project. Findings from this project on third-party responses to deontological rule violations (i.e., homicides) have already been reported [cf. 21]. Here, we focus on third-party judgements of utilitarian transgressions in public health-related scenarios.

### Participants

Participants were recruited in German-speaking countries via advertisements in social networks and invitation emails on mailing lists of the German National Academic Scholarship Foundation and several German universities. A total of *N* = 1004 participants (534 women, 470 men) completed the study. Participants were on average between 22 and 27 years old (*Mdn* ± *QD* = 24.5 ± 2.3, range 18.1–58.0) and primarily German native speakers (97.6%). The sample was characterized by higher school education (99.1% completed the German high-school certificate Abitur) and further academic education. At the time of participation, 62.2% were studying at universities, 31.3% had already graduated from university with a Master’s or Diploma degree, and 5.9% were attending vocational job training. Participants’ specializations encompassed all academic disciplines (including psychology albeit with 7.1% only). The majority of participants described themselves as atheists or agnostics (52.8%), 37.1% as Christians (23.8% Protestants and 12.4% Catholics), and 6.9% as followers of other beliefs, mostly Buddhism or natural religions.

### General procedure

Participants received invitations via email to a two-step online survey [75]. Before the experiment started, participants received information on the study’s general aim and procedure and confirmed their informed, voluntary consent by selecting a checkbox. In the survey’s first session (approx. 20–40 min), participants completed socio-demographic and personality questionnaires. One week later, they received an invitation email for the survey’s second part (approx. 15–30 min). Participants then read short moral dilemmas. After reading each scenario, they reported their emotional responses and judged the behavior of the scenario’s protagonist. The study design was approved by the ethics committee of the Technische Universität Dresden (no. EK241062016).

### Experimental conditions

The moral dilemmas *epidemic* and *vaccine policy* [76, 77] were rephrased from a third-party perspective (for the exact wording see S1 File). In both scenarios, a protagonist has to make decisions during a public health crisis. If the respective protagonist choses the utilitarian option many more will be saved but at the cost of harm to a minority. In *vaccine policy* this harm is accidental, whereas in *epidemic* a deliberate decision would have to be made to exclude certain persons from treatment. Ultimately, both protagonists decided against these actions and choose not to impose mandatory vaccination or treatment restrictions. As a result, these decisions resulted in a higher number of avoidable deaths, which constitutes a violation of utilitarian principles. Contrasting the two scenarios allows examining whether observers are sensitive to the differences of the rejected alternative (accidental vs. deliberate).

### Assessment of observer responses

Each participant read and responded to each scenario (*within*-subject design). The two scenarios (*vaccine policy*, *epidemic*) were part of a larger study consisting of a practice dilemma to familiarize participants with the procedure followed by 15 moral scenarios (see S1 File). The two scenarios in question were not presented directly after another but embedded in the dilemma series also including filler scenarios. Thus, potential direct carry-over effects [78] from *epidemic* to *vaccine policy* or vice versa can be excluded. After reading a scenario, participants reported on 7-point Likert scales ranging from 0 (*not at all*) to 6 (*very intensive*) the intensity to which they felt seven different moral emotions towards the protagonist of the respective scenario. The four emotions (1) anger/outrage, (2) contempt, (3) moral disgust, and (4) disappointment were averaged to *negative/hostile emotions* (Cronbach’s α = .88 for *vaccine policy* and .91 for *epidemic*), whereas (1) comprehensive affection, (2) sympathy, and (3) compassion/pity were averaged to *understanding emotions* (Cronbach’s α = .84 for *vaccine policy* and .82 for *epidemic*). In addition, participants rated on a 7-point Likert scale ranging from 0 (*completely disagree*) to 6 (*completely agree*) the extent to which they considered the protagonist’s decision morally appropriate. Afterwards, participants indicated whether the protagonist should be punished and if so, how long they should be imprisoned. Duration of prison sentences could be freely set with a slider ranging between 0 and 100 years. Other sanction types (e.g., community work or fines) could not be imposed. There was no time limit for reading a scenario or responding to subsequent questions.

### Personality assessment

The Self-Report Psychopathy Scale III [SRP-III; 79] was used to assess the trait-psychopathy facets *callous affect*, *erratic life-style*, and *interpersonal manipulation*. The German Interpersonal Reactivity Index [IRI-SPF; 80] was used to measure the empathy facets *empathic concern*, *perspective taking*, *fantasy*, and *personal distress*. In addition, the altruism scale of the German revised NEO-Personality Inventory [NEO-PI-R; 81] was employed as measure of trait *altruism*. *Neuroticism* was assessed using the respective scale of the German NEO-Five Factor Inventory [NEO-FFI; 82]. The Justice Sensitivity Short Scales [USS-8; 83] served for measuring *justice sensitivity* from the following four social perspectives: victim, perpetrator, beneficiary, and observer. *Obedience to authorities* was assessed using the respective scale of the ALLBUS survey battery [84]. The German version of the Rational-Experiential Inventory [REI; 85] was used to assess the *need for cognition* and *faith in intuition*; and *self-esteem* was measured with the single-item self-esteem scale [SISE; 86].

### Statistical analyses

The assessed personality facets overlap conceptually and empirically. To reduce the number of predictors for subsequent analyses, superordinate personality factors were extracted by exploratory factor analysis (EFA). Data covariance was adequate for conducting a meaningful EFA (Kaiser-Meyer-Olkin measure of sampling adequacy = .770; Bartlett’s Sphericity test: χ^2^(136) = 5373.08, *p* < .001). To maximize the extracted variance a principal component analysis was conducted. Factors with Eigenvalues λ ≥ 1 were extracted and obliquely Promax-rotated (κ = 4, with Kaiser normalization).

Associations between participants’ emotional, cognitive, and punitive responses were analyzed with repeated measure correlations [R package rmcorr; 87]. Using Kendall’s τ correlations, associations between extracted superordinate personality factors and participants’ emotional, cognitive, and punitive responses were examined. *P*-values were Bonferroni-corrected for the number of computed correlations. In addition, mixed effect regressions were conducted using the R package *lme4* [88, with lmerTest; 89], including scenario (*vaccine policy* vs. *epidemic*) as within-subject factor, personality factors as between-subject factors, and all scenario ⨯ personality factor interactions.

Due to the bimodal distribution of imposed punishments, a logistic mixed effect regression was used to analyze whether the (non-)imposition of imprisonment was predicted by the interplay of scenario and personality factors. In separate linear mixed effect regressions, (i) the duration of imposed imprisonments, (ii) the intensity of negative emotions, (iii) the intensity of understanding emotions, and (iv) the perceived moral appropriateness served as dependent variables and were predicted by the interplay of scenario and personality. Note that the analysis of imprisonment durations are based on the *n* = 363 participants who actually imposed punishments in at least one of the two scenarios. *P*-values were Bonferroni-corrected for the number of conducted analyses (α_*crit*_ < 0.01).

A post-hoc sensitivity analysis with G*Power [90] indicated that our sample enabled us to detect within-between interaction effects with a minimal size of η^2^ = .0036–.0052 in a repeated measures ANOVA (α < 0.01, power 1-β = 0.80) depending on the correlation among the scenario conditions (range: 0.10–0.45).

## Results

### Exploratory factor analysis

Personality facets assessed in this study show substantial inter-relations. We conducted an EFA with the aim of reducing the number of predictors for subsequent analyses and avoiding multicollinearity [see also S1 and S2 Files for additional details]. Briefly, the 17 personality facets assembled to five superordinate personality factors explaining 63% of variance. The first factor (PF1) is characterized by high levels of altruism, faith in intuition, the empathy traits fantasy, empathic concern, and perspective taking on the one hand and low levels of victim injustice sensitivity and the psychopathy trait callous affect on the other. The second factor (PF2) consisted of increased neuroticism (i.e., trait anxiety) and personal distress with decreased self-esteem. The third factor (PF3) is characterized by trait psychopathy with lower altruism and increased justice sensitivity from the perpetrator’s perspective. The fourth factor (PF4) marked justice sensitivity from all perspectives (i.e., perpetrator, victim, observer, and beneficiary). The fifth factor (PF5) spanned a continuum from marked faith in intuition, authority obedience, and victim justice sensitivity on the one hand to a high motivation for critical, analytical thinking (NFC) on the other [see S1 and S2 Files for additional details].

### Descriptive statistics and correlation analyses

Overall, punishments and negative emotions were more marked in the *vaccine-policy* scenario compared to *epidemic*, while understanding emotions and moral appropriateness judgments were less pronounced (see Table 1). Table 2 displays bivariate correlations between personality domains and outcome variables.

**Table 1:** Descriptive statistics and correlations of outcome variables (N = 1004).

	Descriptive statistics *Frequency / Med* (*IQR*)		Repeated-measures correlations				
Outcome	Epidemic	Vaccine Policy	1.	2.	3.	4.	5.
1. Imposition of punishment (yes)	112 (11.15%)	302 (30.08%)	–	–	.35***	-.52***	-.50***
2. Duration of imposed punishments (in years)	0.00 (1.51)	11.19 (6.22)		–	.44***	-.59***	-.52***
3. Negative Emotions	1.75 (0.75)	2.00 (1.50)			–	-.39***	-.44***
4. Understanding Emotions	4.00 (1.00)	2.67 (1.67)				–	.76***
5. Appropriateness	4.00 (2.00)	2.00 (2.00)					–

*Note*:

*** *p* < 1.11e-4, two-tailed, Bonferroni-corrected.

**Table 2:** Correlations of experimental outcomes and personality domains (N = 1004).

		*Vaccine Policy*					*Epidemic*				
		Punishment imposition^†^	Imprisonment duration	Negative Emotions	Under-standing Emotions	Appropriate-ness	Punishment imposition^†^	Imprisonment duration	Negative Emotions	Under-standing Emotions	Appropriate-ness
***Vaccine Policy***	Punishment imposition^†^	–									
	Imprisonment duration	–	–								
	Negative emotions	.35***	.26***	–							
	Understanding emotions	-.41***	-.34***	-.00	–						
	Appropriateness	-.47***	-.42***	-.18***	.46***	–					
***Epidemic***	Punishment imposition^†^	.12*	–	.07	-.13**	.03	–				
	Imprisonment duration^#^	–	.12**	.06	-.11**	.01	–	–			
	Negative emotions	.16***	.11**	.43***	.09*	.03	.11*	.09	–		
	Understanding emotions	-.10	.09	.07	.36***	.07	-.38***	-.30***	.07	–	
	Appropriateness	-.16***	-.14***	-.02	.23***	.14***	-.32***	-.26***	-.06	.45***	–
***Personality***	PF1	-.03	-.04	.29***	.24***	.09**	-.07	-.05	.29***	.22***	.15***
	PF2	-.03	-.04	-.02	-.00	.13***	.08	.06	.02	-.04	-.14***
	PF3	.06	.05	-.07	-.16***	-.05	.17***	.10**	-.11***	-.30***	-.08
	PF4	-.00	-.01	.13***	.09**	.06	.01	.00	.16***	.07	-.00
	PF5	-.00	.00	.10***	-.10***	.13***	.39***	.24***	.14***	-.34***	-.16***

*Note*: Kendall’s τ rank correlations,

* *p* < 5.38e-4,

** *p* < 1.08e-4,

*** *p* < 1.08e-5,

two-tailed, Bonferroni-corrected.

^†^ As punishment imposition is binary (no vs. yes), point-biserial correlations are reported.

PF1 (other-oriented empathy, altruism, faith in intuition, low callous affect); PF2 (anxiety, personal distress, low self-esteem); PF3 (trait psychopathy, low perpetrator justice sensitivity); PF4 (justice sensitivity); PF5 (faith in intuition, victim justice sensitivity, obedience to authorities, low need for cognition).

### Regression analyses

**Fig 1:**
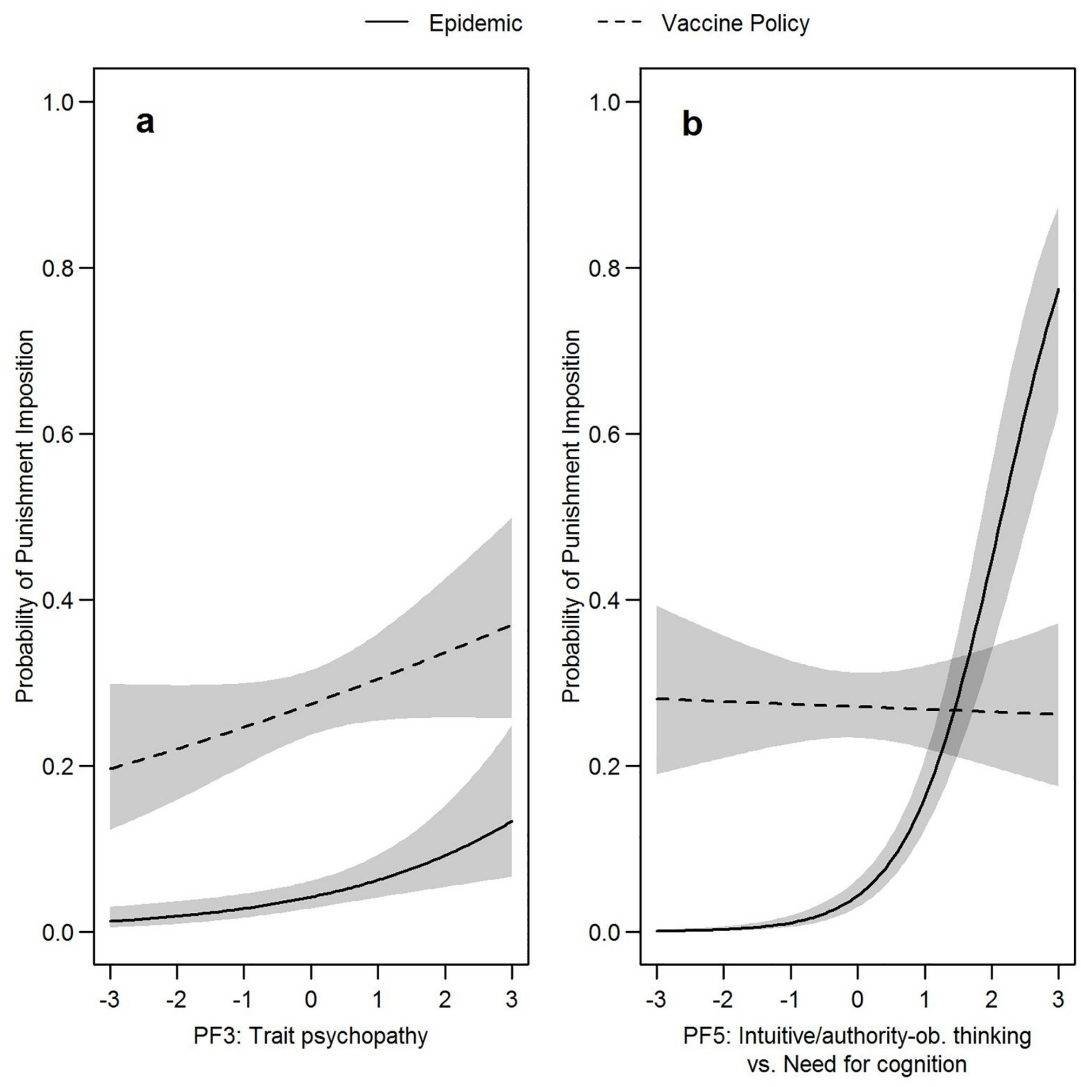
Visualization of the results of the mixed logistic regression. Trend lines illustrate the predicted probability of punishment imposition in the two scenarios *epidemic* (deliberate harm) and *vaccine policy* (accidental harm) depending on observers’ personality differences. Shaded areas represent 95% confidence intervals.

**Fig 2:**
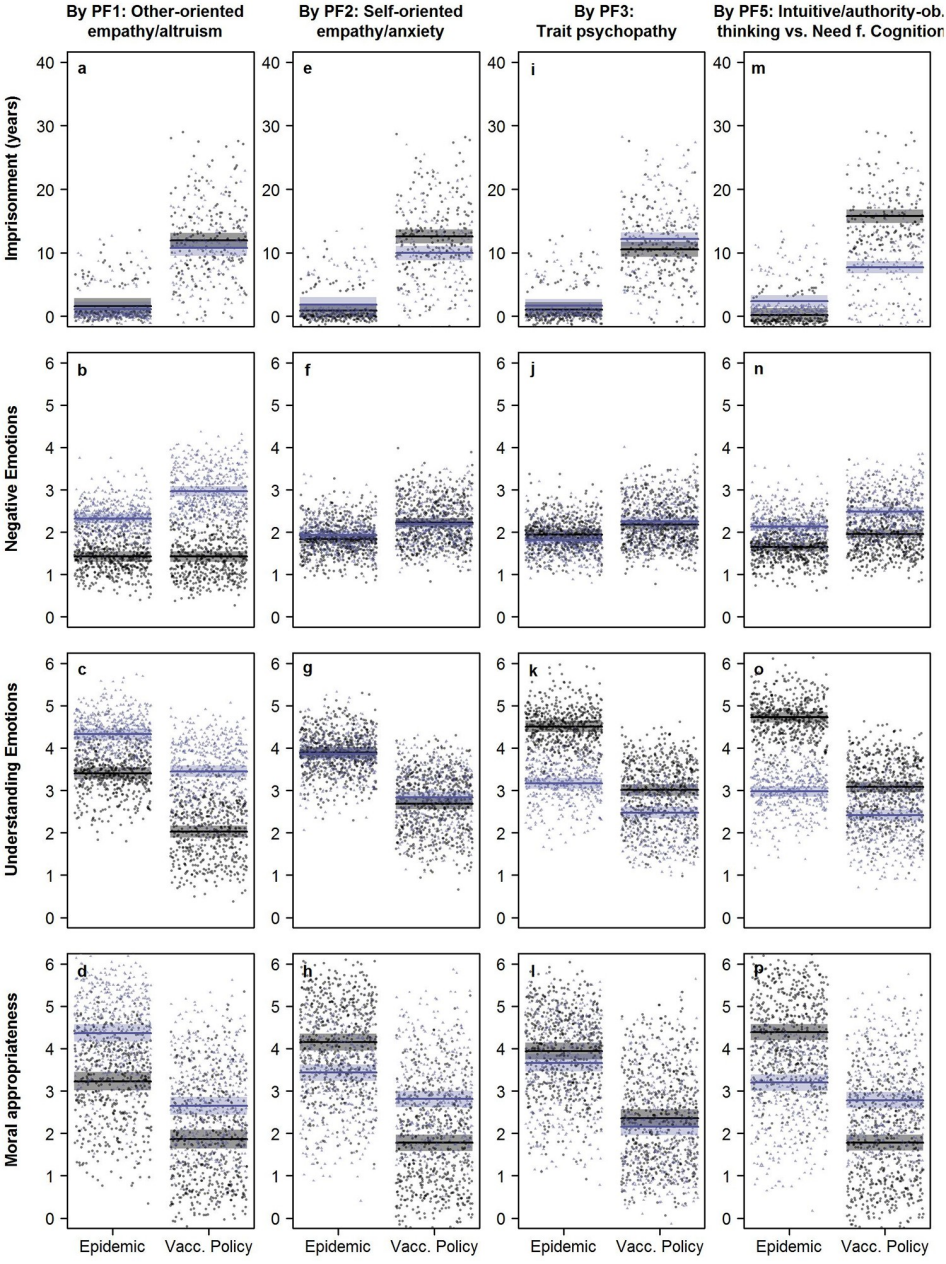
Visualization of the results of the linear mixed regressions. The graphs show punitive, emotional, and cognitive responses (see columns) to harmful omissions protecting a minority from deliberate harm (*epidemic*) or accidental harm (*vaccine policy*) depending on observers’ personality differences (see rows). Lower scores (*M*-2*SD*) of the respective personality factor are indicated by grey color and higher scores (*M*+2*SD*) by black color. Shaded areas represent 95% confidence intervals.

Tables 3 and 4 summarize the results of the logistic and linear mixed effect regressions analyzing emotional, cognitive, and punitive reactions to the respective scenario (*vaccine policy* vs. *epidemic*) depending on personality differences. Notably, 69.9% (*vaccine policy*) and 88.8% (*epidemic*) of our participants indicated that the respective protagonist should not be punished at all. The logistic regression (Table 3) showed that punishment was 7.8 times more likely when opting to favor the majority would have caused *accidental* harm (*vaccine policy*) compared to if this would have led to *deliberate/targeted* harm of a minority (*epidemic*). If a prison sentence was assigned, imprisonment durations for *vaccine policy* were on average 10.4 years longer than for *epidemic* (Table 3). Correspondingly, third parties felt more negative and less understanding emotions in the *vaccine-policy* scenario. They also judged the inaction in this dilemma as less morally appropriate compared to *epidemic* (Table 4). This general pattern was further modified by personality (see Figs 1 and 2) whose effects are detailed in the following chapter.

**Table 3:** Results of logistic and linear mixed effect regressions for punishment imposition and severity.

	Logistic mixed effect model for punishment imposition (no/yes, *N* = 1004)						Linear mixed effect model for the duration of imposed imprisonment (in years, *n* = 363^#^)					
Predictors	*B*	*SE*	*z*	*p*	OR	90%CI(OR)	*b*	*SE*	β	*t*	*p*	_ $\eta_{p}^{2}$ _
Intercept	-2.01	0.13	-15.66	**< .001**			6.49	0.18		36.01	**< .001**	
Scenario	2.06	0.19	11.05	**< .001**	7.83	[5.76, 10.63]	10.38	0.36	.74	28.79	**< .001**	.699
PF1	-0.06	0.09	-0.71	.475	0.94	[0.82, 1.08]	-0.21	0.20	-.03	-1.06	.290	.003
PF2	-0.02	0.08	-0.31	.757	0.98	[0.86, 1.11]	-0.21	0.18	-.03	-1.19	.236	.004
PF3	0.28	0.08	3.57	**< .001**	1.33	[1.16, 1.51]	0.28	0.18	.04	1.53	.126	.007
PF4	0.07	0.08	0.85	.397	1.07	[0.93, 1.23]	-0.24	0.19	-.04	-1.26	.209	.004
PF5	0.71	0.09	8.15	**< .001**	2.04	[1.77, 2.36]	-0.73	0.16	-.12	-4.62	**< .001**	.057
Scenario ⨯ PF1	-0.01	0.16	-0.05	.963	0.99	[0.76, 1.29]	-0.17	0.40	-.01	-0.43	.667	.001
Scenario ⨯ PF2	-0.13	0.15	-0.89	.373	0.88	[0.69, 1.12]	-0.89	0.36	-.07	-2.50	.013	.017
Scenario ⨯ PF3	-0.27	0.15	-1.88	.061	0.76	[0.60, 0.97]	0.26	0.36	.02	0.72	.473	.001
Scenario ⨯ PF4	0.00	0.16	-0.02	.984	1.00	[0.77, 1.29]	-0.26	0.39	-.02	-0.67	.503	.001
Scenario ⨯ PF5	-1.46	0.17	-8.79	**< .001**	0.23	[0.18, 0.31]	-2.56	0.32	-.21	-8.08	**< .001**	.155
Overall model statistic	χ^2^(11) = 296.97, *p* < 2.2e-16***, *R*^2^ = .473, *d*’ = .823						*F*(11, 610.07) = 82.69, *p* < 2.2e-16***, *R*^2^ = .557					

*Note*:

^#^ Subsample of participants who assigned an imprisonment in at least one of the two scenarios.

The effect size measure $\eta_{p}^{2}$ indicates how much variance is accounted for by the predictor when controlling for any other predictors. $\eta_{p}^{2}$ can only be approximated in multilevel models, and, in the present case, it is based on Type III Wald *F* tests with degrees of freedom approximated with the Kenward-Roger method.

**Table 4:** Results of linear mixed effect regressions for observers’ emotional and cognitive responses (N = 1004).

	Negative Emotions [0,6]						Understanding Emotions [0,6]						Perceived moral appropriateness [0,6]					
Predictors	*b*	*SE*	β	*t*	*p*	_ $\eta_{p}^{2}$ _	*b*	*SE*	β	*t*	*p*	_ $\eta_{p}^{2}$ _	*b*	*SE*	β	*t*	*p*	_ $\eta_{p}^{2}$ _
Intercept	2.04	0.02		102.85	**< .001**		3.26	0.02		140.41	**< .001**		3.02	0.03		89.81	**< .001**	
Scenario	0.32	0.02	.19	13.27	**< .001**	.150	-1.08	0.03	-.46	-34.47	**< .001**	.544	-1.46	0.05	-.46	-27.52	**< .001**	.431
PF1	0.31	0.02	.37	13.23	**< .001**	.149	0.29	0.03	.25	10.86	**< .001**	.106	0.24	0.04	.15	6.14	**< .001**	.036
PF2	0.01	0.02	.01	0.40	.693	.000	0.01	0.02	.01	0.58	.564	.000	0.04	0.04	.02	1.09	.276	.001
PF3	-0.01	0.02	-.01	-0.33	.745	.000	-0.23	0.03	-.20	-9.17	**< .001**	.078	-0.06	0.04	-.04	-1.62	.105	.003
PF4	0.03	0.02	.03	1.24	.216	.002	0.00	0.03	.00	-0.01	.991	.000	-0.04	0.04	-.02	-0.96	.335	.001
PF5	0.13	0.02	.15	6.20	**< .001**	.037	-0.30	0.02	-.26	-12.70	**< .001**	.139	-0.02	0.03	-.01	-0.69	.491	.000
Scenario ⨯ PF1	0.16	0.03	.10	5.63	**< .001**	.031	0.13	0.04	.05	3.43	**.001**	.012	-0.09	0.06	-.03	-1.43	.153	.002
Scenario ⨯ PF2	-0.03	0.03	-.02	-1.29	.198	.002	0.04	0.03	.02	1.33	.183	.002	0.44	0.06	.14	7.84	**< .001**	.058
Scenario ⨯ PF3	0.05	0.03	.03	1.95	.051	.004	0.20	0.03	.09	5.85	**< .001**	.033	0.02	0.06	.01	0.35	.729	.000
Scenario ⨯ PF4	0.01	0.03	.00	0.22	.829	.000	0.02	0.04	.01	0.43	.667	.000	-0.01	0.06	.00	-0.18	.859	.000
Scenario ⨯ PF5	0.01	0.03	.01	0.36	.722	.000	0.27	0.03	.11	8.42	**< .001**	.066	0.55	0.05	.17	10.04	**< .001**	.092
Overall model statistic	*F*(11, 1714.44) = 47.47, *p* < 2.2e-16***, *R*^2^ = .569						*F*(11, 1714.44) = 169.96, *p* < 2.2e-16***, *R*^2^ = .650						*F*(11, 1714.44) = 92.43, *p* < 2.2e-16***, *R*^2^ = .453					

*Note*: The effect size measure $\eta_{p}^{2}$ indicates how much variance is accounted for by the predictor when controlling for any other predictors. $\eta_{p}^{2}$ can only be approximated in multilevel models, and, in the present case, it is based on Type III Wald *F* tests with degrees of freedom approximated with the Kenward-Roger method.

### Main and interaction effects of observer personality

Participants with more marked PF1 characteristics (other-oriented empathy, altruism, faith in intuition, low callous affect) felt more intense negative (Fig 2b) as well as understanding emotions (Fig 2c) in both scenarios. However, their negative and understanding emotions were particularly increased in *vaccine policy*. In both dilemmas, they judged the rejection of the utilitarian option as more morally appropriate (Fig 2d) than participants with lower PF1 levels. No association was found between PF1 and the probability or severity of punishment (Figs 1a and 2a).

PF2 characteristics (anxiety, personal distress, low self-esteem) were not associated with differences in punishment probability or severity (Fig 2e). However, there was a scenario ⨯ PF2 interaction on moral appropriateness ratings. The response pattern reveals that low compared to high PF2 characteristics lead to higher moral appropriateness ratings in *epidemic* while the opposite was true for *vaccine policy*. As Fig 2h shows, there is a considerable difference in appropriateness ratings in the two dilemmas for participants with less marked PF2 characteristics. Contrariwise, the appropriateness ratings of high PF2 individuals were similar across the two dilemmas. Thus, while both PF2 groups rated the decision in *epidemic* as more appropriate than the decision in *vaccine policy*, the discrepancy was larger for low PF2 individuals. PF2 was not associated with differences in negative and understanding emotions (Fig 2f and 2g).

Observers with marked PF3 characteristics (trait psychopathy, low perpetrator justice sensitivity) generally reported less understanding emotions for the protagonists in both scenarios with understanding emotions being particularly decreased in *epidemic* (Fig 2k). PF3 was not associated with differences in negative emotions (Fig 2j) or moral appropriateness ratings (Fig 2l). However, PF3 was associated with an increasing probability of punishment in both scenarios (Fig 1a), but did not account for differences in the severity of imposed punishments (Fig 2i).

There were no significant main or interaction effects for PF4 (other-oriented justice sensitivity).

Participants with higher PF5 levels (faith in intuition, victim justice sensitivity, obedience to authorities, low NFC) felt more negative emotions in both scenarios (Fig 2n). They also felt less understanding emotions which was especially evident in *epidemic* (Fig 2n). There was also a PF5 × dilemma interaction effect on moral appropriateness ratings. In *epidemic*, participants with lower PF5 levels judged the inaction of the protagonist as more morally appropriate compared to participants with higher PF5 levels. However, in *vaccine policy*, lower PF5 levels were associated with less perceived moral appropriateness of the inaction in question. Intriguingly, judgements of appropriateness in both dilemmas did not differ by much in participants with high PF5 levels while participants with low PF5 levels rated appropriateness in the two scenarios very differently (Fig 2p). Higher PF5 levels were also accompanied by a higher probability to punish the omission of an intervention that would have caused deliberate harm (*epidemic*) (Fig 1b). PF5 main and interaction effects were also found on the duration of assigned imprisonments. Observers with low PF5 levels differentiated strongly between the two dilemmas and imposed harsher sanctions specifically on the protagonist of the *vaccine-policy* scenario (Fig 2m). Contrariwise, observers with high PF5 levels assigned similar punishments in both scenarios.

## Discussion

Appropriate reactions by third parties to norm transgressions are vital for social functioning [9, 55]. Here, we explored third-party responses to public health-related decisions that, while protecting the rights and lives a minority ultimately caused more net harm and thus violated utilitarian rules.

### Role of dilemma content features

Our findings in a sample of about 1,000 adults emphasized the importance of specific features of moral scenarios. More precisely, the question whether the avoided harm to a minority would have been *accidental* (*vaccine policy*) or *deliberate/targeted* (*epidemic*) proved to be decisive. Importantly, in *vaccine policy* it is unknown, who is part of the minority group at risk to die due to allergies to the vaccine, and thus, individuals at risk cannot be reliably identified prior to vaccination. Contrariwise, in *epidemic*, the members of the minority (X type) would have to be identified with the aim to be deliberately and actively excluded from treatment and thus condemned to die. Avoiding *accidental* harm to a minority (*vaccine policy*) elicited more marked negative emotions and less understanding emotions. Consistently, it was also rated as less morally appropriate and was punished more frequently and more severely. However, it should be noted that a substantial portion of the participants did *not* punish the protagonist in either scenario at all (69.9% and 88.8%, respectively). Thus, participants’ responses to both scenarios also reflect the fact that the protagonists’ choice against the respective utilitarian option was not simply perceived as “immoral” even though it resulted in extensive harm. Furthermore, our results are also in line with findings on the interplay of distributive and procedural fairness. Generally, unsatisfactory or unfair outcomes have been found to be more acceptable when they result from fair procedures [overview: 91]. Since in *vaccine policy* members of the minority cannot be identified beforehand, their random sacrifice might constitute a rather fair procedure compared to the deliberate selection necessary in *epidemic*. Thus, opting against the random sacrifice of an unidentifiable minority in *vaccine policy* should be perceived as more punishment-deserving according to research into the effects of fair vs. unfair procedures, which is exactly what we found in our sample.

While both *epidemic* and *vaccine policy* are oversimplified fictional scenarios, similar public health-related issues might occur in real life and could potentially affect entire societies. Without doubt, various conflicting action-guiding ethical motives need to be considered and integrated by policy makers when deciding on public health scenarios. Unspecific ‘deontic rules’ fall short as an ethical justification of non-action especially with regard to scenarios of more realistic complexity, and accordingly, medical ethics professionals consider and integrate a number of relevant ethical positions as guiding principles in real-world public health scenarios [see 92–94, for a detailed discussion]. However, despite their simplification, responses of third parties to public health dilemmas provide some indication into potential reactions of the public to real events.

While this study was conducted before the start of the COVID-19 pandemic, the latter emphasizes the relevance of our findings. At present (autumn 2022), the further development of worldwide infection rates cannot be reliably predicted, nor can the death toll or whether the various vaccination efforts will eventually ensure herd immunity. Still, we do not wish to overemphasize similarities between the COVID-19 pandemic and some of the features of the dilemmas used here. However, there has been at least one incident in the past that represents, essentially, a real-life counterpart to the *vaccine policy* scenario illustrating the crucial role of the public (i.e., third parties). In contrast to the *vaccine policy* scenario in our study, authorities did opt for the utilitarian approach. Fortunately, the feared millions of deaths did not occur. Unfortunately, the respective vaccination program was nevertheless not exactly a success story: In the fall of 1976, an unprecedented nation-wide vaccination program against a new influenza virus started in the United States of America [95]. The virus in question had been identified as a descendant of the 1918 strain that caused the Spanish Influenza [95], which had an estimated death toll of 50–100 million worldwide [96]. However, the dangerousness of the new virus was still uncertain. The risk of a new pandemic was judged to be small, but not zero [95]. Furthermore, massive inoculation programs come not only with massive financial and organizational costs, they come with the potential risk of a considerable number of people affected by side effects. Experts also warned of the risk that unrelated issues that simply coincided with the inoculation might nevertheless in the public’s mind be seen as caused by the vaccine [e.g., 97]. Thus, a timely decision had to be made in the face of many uncertainties. The program finally started in October 1976 and almost immediately reports followed of serious complications allegedly caused by the vaccine [95]. Confusion about and distrust in the vaccine grew in the public. Meanwhile, the feared deadly flu outbreak did not manifest. In the end, the program was prematurely terminated in December 1976 [for a detailed history see also 98]. Since the anticipated benefit to the majority was not there, the program was seen as only causing harm to a minority. In the aftermath, communication with and involvement of the public (i.e., third parties) was identified as one crucial factor for the failed implementation [95, 98].

### Role of personality for moral emotions

Aside from situational features like miscommunication with the public, differences in personality might affect third parties’ emotional, cognitive, and punitive responses to perceived moral rule violations. In this study, negative moral emotions like anger, contempt, disgust, and disappointment were associated with personality aspects that clustered in PF1 and PF5. Thus, negative emotions were more pronounced in participants who scored higher in empathy, altruism, faith in intuition, victim justice sensitivity, obedience to authorities, and lower in NFC and callous affect. While the effect was present in both dilemmas, it was particularly strong in the case of *accidental* harm (*vaccine policy*). Intriguingly, increased *understanding* emotions like compassion, comprehension, and sympathy towards a dilemma’s protagonist were also associated with some of these traits, i.e., empathy, altruism, faith in intuition, and low callous affect (PF1). Thus, this group of traits resulted generally in more emotionally charged responses reflecting righteous anger in reaction to the harm caused but also consideration for the predicament of the protagonist. As Tangney et al. [99] summarized, empathy is an emotional process which affects various aspects of moral behavior including feelings of concern, helping behavior, and inhibited aggression. As our findings indicate, empathic tendencies of concern and sympathy for others are not necessarily limited to the victims of harmful acts but may extend to decision makers in difficult situations.

Contrariwise, higher scores in psychopathy and low perpetrator justice sensitivity (PF3) as well as increased scores in faith in intuition, victim justice sensitivity, obedience to authorities, but low NFC (PF5) were associated with *less* understanding emotions in general and for *epidemic* in particular. Hence, unlike PF1, the emotional pattern linked to higher PF5 scores was marked by enhanced negative and reduced understanding emotions, which is in line with findings reporting increased negative emotions in moral dilemma situations in authoritarian individuals [61, 64]. As for PF3, compassion and sympathy for others show distinct negative associations with psychopathy in general [40, 100]. Furthermore, the second component of PF3 (i.e., low perpetrator justice sensitivity) specifically reflects a less pronounced ability and/or willingness to consider motives and reasons for the actions of a transgressor [57].

### Role of personality for moral appropriateness ratings

In addition to moral emotions, moral appropriateness ratings were also modulated by personality factors. Individuals with higher PF1 scores (empathy, altruism, faith in intuition and low callous affect) rated the protagonists’ decision *against* the utilitarian option in both scenarios as more morally appropriate. This result is in line with findings that show negative associations between empathy and utilitarian tendencies [48, 101]. Furthermore, there were interaction effects on moral appropriateness ratings between dilemma type and two additional personality factors: PF2 (anxiety, personal distress, low self-esteem) and PF5 (faith in intuition, victim justice sensitivity, obedience to authorities, low NFC). Participants with higher PF2 and PF5 scores rated moral appropriateness in response to the two dilemmas comparatively similar. Contrariwise, ratings of participants with low PF2 and PF5 scores differed considerably, indicating a more nuanced approach when assessing different moral decisions. In particular the findings for PF5 are in line with previous results since obedience to authorities (PF5) is associated with a lesser consideration of context and a preference for fixed rules [7, 62, 63]. In the third-party perspective investigated here, similar moral appropriateness ratings across the two scenarios of participants with higher PF5 scores also reflect less consideration for the differences between the dilemma characteristics. One other important component of PF5, i.e., low NFC, is by definition characterized by less interest in and enjoyment of effortful cognition [102], which might have also contributed to the results pattern. Intriguingly, a similar pattern to PF5 was observed for PF2 (anxiety, personal distress, low self-esteem). Anxiety affects cognitive processes by biasing attention towards threat-related information and facilitating negative interpretations of ambiguous stimuli [review: 103]. Furthermore, anxiety has been suggested to diminish cognitive resources [104] which might explain the less differentiated ratings.

### Role of personality for punishment of perceived transgressions

Finally, the likelihood to impose sanctions as well as the severity of punishment depended on personality. Higher scores in psychopathy and low perpetrator justice sensitivity (PF3) were associated with an increased willingness to punish in both dilemmas. The finding that individuals with higher psychopathy scores (PF3) punish more frequently corresponds with their reported preference for utilitarian rules [45–47], which are violated in both scenarios. Furthermore, some of the key characteristics of psychopathy are a distinct need for social dominance and a grandiose sense of self [100], both of which might lead to a greater willingness to punish when given the opportunity [100]. Another relevant feature might also be their decreased aversion to perform harmful acts, e.g., to punish others [42]. In addition, higher PF5 scores (faith in intuition, victim justice sensitivity, obedience to authorities, low NFC) were linked to a higher likelihood to impose punishments in *epidemic*. This is in line with findings that link authoritarianism to increased punitiveness [105, 106]. Furthermore, victim justice sensitivity (another component of PF5) refers to one’s own perceived disadvantages but also to the tendency to retaliatary responses [57]. Furthermore, it has been linked to proactive, reactive, and relational aggression [107] as well as antisocial and egotistic behavior [108]. Moreover, when imposing sanctions, observers with higher PF5 scores did not differentiate very clearly between *epidemic* and *vaccine policy* regarding the penalty level. Individuals’ high in intuitive/authority-obedient thinking have been suggested to be less willing and/or able to deliberate the situational setting of a (moral) problem in all its complexity or to integrate more than one perspective on it. Rather, they rely on applying situationally invariant deontic rules when making or judging moral decisions [7, 61–63], which might be reflected in our findings. Conversely, observers with low PF5 scores (i.e., high NFC) clearly differentiated between the scenario conditions and, accordingly, imposed considerably longer imprisonments specifically in the *vaccine-policy* condition. Correspondingly, previous studies found that NFC promotes a preference for utilitarian decisions [7, 67, 109]. The latter was explained by a higher individual ability and/or willingness to invest cognitive efforts to identify, deliberate, and weigh motives, reasons, and situational constraints when making decisions or judging others’ actions [7, 66, 110].

### Third-party judgements in summary

In sum, our exploration of third-party judgements on moral decisions affecting large parts of a society revealed distinct responses, depending on whether *accidental* or *deliberate* harm to a minority was avoided. However, response patterns to the presented utilitarian rule violations were further modulated by various aspects of personality underscoring the importance of inter-individual differences. While PF1 (empathy, altruism, faith in intuition, and low callous affect) modulated moral emotions as well as perceived appropriateness it did not affect punishment. Conversely, PF2 (anxiety, personal distress, low self-esteem) did not affect emotional responses and punishment but modulated appropriateness ratings, while PF3 (higher psychopathy, low perpetrator justice sensitivity) influenced selectively understanding emotions and the willingness to punish. The most comprehensive effects were arguable found for PF5 (faith in intuition, victim justice sensitivity, obedience to authorities, low NFC) which affected all assessed responses, i.e., moral emotions, appropriateness ratings, and punishment. Intriguingly, PF4 (justice sensitivity) was neither associated with emotional, cognitive or punitive responses which is surprising given that previous studies have linked the trait to responses to moral dilemmas [28, 41, 49, 58]. However, it should be noted that two components of justice sensitivity, i.e., perpetrator and victim justice sensitivity, also loaded on PF3 and PF5, respectively, and exerted some influence on moral judgements.

### Limitations

The study extensively explored associations between observers’ personality and their emotional, cognitive, and punitive reactions to utilitarian rule violations in a large sample (*N* = 1,004) that allowed for detecting even small situation-personality interactions. However, as moral judgments might differ depending on participants’ educational, religious, and cultural background, our results’ generality is limited to predominantly young, well-educated, atheist or Christian adults from Western Europe.

Additional limitations of our study include dilemmas features. Although widely employed in experimental research, the ecological validity of moral dilemmas is nevertheless debatable. Furthermore, these moral scenarios have in part ambiguous content features that might result in different interpretations and thus different responses. Aggregating various moral dilemmas within one condition category has been criticized due to the high content heterogeneity of frequently used moral dilemmas [72]. For a more detailed discussion of potential effects of varying dilemma features and differences in participants’ interpretation of moral dilemmas see also Behnke et al. [21]. In order to avoid unwanted error variance, we limited this study to two scenarios contrasting one clearly defined condition difference. Still, there are additional varying features in both dilemmas, which limit direct comparisons between them. Future research is needed to investigate the interplay between personality and other situational or motivational conditions of moral-ethical dilemma scenarios. Furthermore, the dilemmas focus closely on public health-related issues, which limits the generalizability of our results. Additional research is also needed to investigate ethical motives behind real-life decisions need more in-depth investigation since rather unspecific ‘deontic’ rules come up short as explanations for choices in more complex and realistic scenarios. In addition, recently published studies investigating moral actions in virtual reality [VR; 111, 112] reported very different response patterns than those usually found in self-report assessments of moral judgments like the one used here. Generally, participants responded in virtual environments with greater utilitarian actions. Although the VR-studies used a different dilemma (i.e., *footbridge*), one might speculate whether the high percentage of participants who in our study chose *not* to punish utilitarian rule transgressions and thus endorsed the deontological decision made by the protagonist, would also do so in VR—or in real life.

Finally, while we measured emotional, cognitive, and punishing responses after each scenario, we had to limit this assessment. Given the substantial time needed to complete the entire survey, the assessment of emotional responses (e.g., feelings of anger or disgust) had to be restricted to one item for each moral emotion (see also S1 File for more details). Using even just a short questionnaire to assess every moral emotion of interest after each moral dilemma would have been unfortunately very impractical.

## Conclusions

In our exploratory study, most third-party observers did *not* punish decisions that resulted in tremendous collective harm when safeguarding the rights (and lives) of a minority was the presumed reason behind a protagonist’s decision to disregard the rights of the majority. However, depending on whether the (avoided) violation of minorities would have been *accidental* or *deliberate/targeted*, third parties’ responses varied. While almost a third of the participants indicated that avoiding *accidental* harm of a minority at high costs to the majority should be punished, only 11.5% were willing to punish decisions that avoided *deliberate* harm. Furthermore, key personality traits were associated with cognitive, emotional, and punitive responses to the selected moral dilemmas. Given the importance of third parties in maintaining moral standards in society and in keeping social life functioning, the results provide insights into the complex mechanisms behind differences in moral responses of uninvolved observers.

## Supporting information

S1 File Study material. (PDF)

S2 File Exploratory factor analysis. (PDF)
