# Supplementary material for: The needs of the many: Exploring associations of personality with third-party judgments of public health-related utilitarian rule violations
Source: PLoS One. 2023 Apr 21;18(4):e0284558. doi: 10.1371/journal.pone.0284558 (PMC10121057; doi:10.1371/journal.pone.0284558)
Supplement: S2 File — (PDF) [file pone.0284558.s002.pdf]

The needs of the many: Exploring associations of personality with third-party judgments of public health-related utilitarian rule violations

- Supplemental Material 2 –

Alexander Behnke, Diana Armbruster, Anja Strobel

Exploratory factor analysis (EFA)

Supplementary Table 1. Results of exploratory factor analysis

	Extracted Personality Factor					Communalities
**Entered Personality Facets**	***PF1***	***PF2***	***PF3***	***PF4***	***PF5***	***h²***
Altruism (NEO-PI-R)	**.478**	-.246	**-.441**	.000	.149	.651
Neuroticism (NEO-FFI)	.187	**.790**	.155	.159	.145	.759
Victim justice sensitivity (USS-8)	**-.340**	-.024	.094	**.665**	**.435**	.644
Observer justice sensitivity (USS-8)	.111	-.019	.022	**.793**	-.095	.683
Beneficiary justice sensitivity (USS-8)	.045	.117	-.021	**.707**	-.165	.576
Perpetrator justice sensitivity (USS-8)	.065	.020	**-.426**	**.436**	-.243	.564
Faith in intuition (REI)	**.458**	-.327	.193	.066	**.563**	.656
Need for cognition (REI)	.096	-.288	.145	.149	**-.757**	.696
Obedience to authorities	-.049	.141	-.141	-.125	**.694**	.509
Self-esteem	.002	**-.798**	.124	-.062	-.055	.706
Interpersonal manipulation (SRP-III)	.049	-.020	**.845**	.027	-.007	.678
Callous affect (SRP-III)	**-.410**	.023	**.605**	-.059	-.105	.751
Erratic life-style (SRP-III)	.261	-.073	**.762**	.047	-.176	.511
Fantasy (IRI-SPF)	**.807**	.234	.301	-.015	.020	.560
Empathic concern (IRI-SPF)	**.722**	.191	-.108	.138	-.012	.708
Perspective taking (IRI-SPF)	**.645**	.043	.025	-.083	-.282	.441
Personal distress (IRI-SPF)	.181	**.781**	-.047	-.090	.125	.628
	PF1	***PF2***	***PF3***	***PF4***	***PF5***	Total
Factor Eigenvalues (λ)	3.94	2.67	1.57	1.47	1.08	
Variance proportion (%) per factor	23.18	15.69	9.24	8.63	6.33	63.07
**Domain correlation matrix**	***PF1***	***PF2***	***PF3***	***PF4***	***PF5***	
PF1	1.00	-.11	-.37	.37	.02	
PF2		1.00	-.07	.20	.13	
PF3			1.00	-.23	.12	
PF4				1.00	.11	
PF5					1.00	

Note: Results of the EFA have already been reported in Behnke, Strobel, Armbruster (2020)
